# Assessing the use of mobile phone data to describe recurrent mobility patterns in spatial epidemic models

**DOI:** 10.1098/rsos.160950

**Published:** 2017-05-17

**Authors:** Cecilia Panigutti, Michele Tizzoni, Paolo Bajardi, Zbigniew Smoreda, Vittoria Colizza

**Affiliations:** 1 Dipartimento di Fisica, Università degli Studi di Torino, via Giuria 1, Torino 10125, Italy; 2 ISI Foundation, via Alassio 11/C, Torino 10126, Italy; 3 Aizoon Technology Consulting, Str. del Lionetto 6, Torino, Italy; 4 Sociology and Economics of Networks and Services Department, Orange Laboratories, Issy-les-Moulineaux, France; 5 Sorbonne Universités, UPMC Univ Paris 06, INSERM, Institut Pierre Louis d’Epidémiologie et de Santé Publique (IPLESP, UMR–S 1136), Paris, France

**Keywords:** epidemic modelling, infectious diseases, mobile phones, spatial epidemiology

## Abstract

The recent availability of large-scale call detail record data has substantially improved our ability of quantifying human travel patterns with broad applications in epidemiology. Notwithstanding a number of successful case studies, previous works have shown that using different mobility data sources, such as mobile phone data or census surveys, to parametrize infectious disease models can generate divergent outcomes. Thus, it remains unclear to what extent epidemic modelling results may vary when using different proxies for human movements. Here, we systematically compare 658 000 simulated outbreaks generated with a spatially structured epidemic model based on two different human mobility networks: a commuting network of France extracted from mobile phone data and another extracted from a census survey. We compare epidemic patterns originating from all the 329 possible outbreak seed locations and identify the structural network properties of the seeding nodes that best predict spatial and temporal epidemic patterns to be alike. We find that similarity of simulated epidemics is significantly correlated to connectivity, traffic and population size of the seeding nodes, suggesting that the adequacy of mobile phone data for infectious disease models becomes higher when epidemics spread between highly connected and heavily populated locations, such as large urban areas.

## 1. Introduction

In the last decade, the analysis of individual call detail record (CDR) extracted from mobile phone data has provided numerous insights into the quantitative patterns that characterize human everyday life [1]. In particular, mobile phone data have proved to be an excellent source to describe human movements at the finest scales, providing unprecedented details on individual mobility patterns and highlighting some universal features, such as the high degree of predictability of individual trajectories which coexists with strong heterogeneities of collective patterns [2–5].

The availability of human mobility data at such high resolution has impacted several research fields, ranging from urban planning to social sciences [6–11], but one of its most successful applications has undoubtedly been the spatial epidemiology of infectious diseases [12–18]. A detailed description of human mobility is important for characterizing and forecasting the spatial and temporal spread of infectious diseases [19] and human movement data have become an essential ingredient for most spatial epidemic models, both at global [20–22] and national or continental scale [23–25]. The urgent need for accurate mobility data to inform epidemic models has been recently spotlighted during the 2014 West Africa Ebola virus disease (EVD) outbreak [26,27]. Other recent global public health threats, such as the 2013 MERS-CoV outbreak and the 2016 Zika virus outbreak, have called for a prompt characterization of human movements originating from the affected areas to properly inform modelling efforts and assess the risk of importation to the rest of the world [28,29].

Although its importance is widely recognized, an accurate description of human mobility in a given country or region is often challenged by several issues. First, the lack of reliable official data sources, especially in low-income countries and regarding short-range mobility [21]. Second, the limited availability of alternative data sources such as call detail record data owing to privacy and ethical concerns [30]. Finally, the limited generalizability of mobility models [31] whose performance can significantly vary depending on the specific geographical setting and modelling assumptions [13,32], and whose use can be hindered in the absence of good calibration data. Furthermore, epidemic modelling results can be sensitive to choices in the parametrization of mobility models, accuracy in the definition of initial conditions [33] and to the type of mobility under study [34].

All the above uncertainties call for a quantitative assessment of using different proxies to describe human movements in spatial epidemic models, to better understand how modelling results are affected by limitations inherent to the various available data sources. Among the vast literature on the use of mobile phone data, there are a few studies presenting a side-by-side comparison of different proxies for human mobility with applications to infectious disease modelling, especially considering mobile phone data [35–37]. These studies pointed out some important differences in estimates of human movements from mobile phone records when compared with official surveys or mobility models. More specifically, travel volumes tend to be larger when measured by CDRs than by surveys or census. By contrast, the overall network topology is usually well captured by mobile phone data, with differences mainly affecting less connected or less populated areas [36].

In this study, we present an extensive side-by-side comparison of simulated epidemics in France based on two commuting networks: one extracted from an official census survey and one from a large-scale mobile phone dataset. We have previously examined the two networks in terms of their statistical features, comparing their topology and distributions of travel flows, and found a good statistical agreement between the two [36]. By contrast, previous results based on simulated epidemics on the two networks have shown that simulation outcomes may vary substantially when using one dataset or the other, depending on the specific outbreak location and disease parameters [36]. Here, we thoroughly assess the adequacy of the mobile phone network to match epidemic patterns that have been generated by simulations using the census data. Our goal is to test the goodness of the mobile phone mobility network to replace the census survey mobility network, which is explicitly assumed to be the best representation of commuting patterns in France. To this aim, we compare the spatio-temporal properties of simulated outbreaks originating from every possible seed of the mobility networks and quantify their similarity in terms of the epidemic invasion tree and arrival time of first infection. We identify the features characterizing the outbreak seed nodes that best correlate the similarity between epidemic patterns and discuss how these results can help to assess the adequacy of mobile phone data to describe recurrent mobility patterns in spatial epidemic models.

## 2. Methods

### 2.1. Commuting networks

We compared simulated epidemics based on the movements of French commuters extracted from two different data sources: the *census* commuting network and the *mobile phone* commuting network.

The census commuting network is extracted from the database of the French National Institute of Statistics and Economic Studies [38], reporting the results of the 2007 National Census Survey. Commuting data are collected each year by the National Census Survey, which samples all the residents in municipalities with less than 10 000 inhabitants and about 8% of the households in the other municipalities. Then, a full database is generated by assembling five surveys conducted on five consecutive years, resulting in an overall coverage of about 40% of the population in municipalities with more than 10 000 inhabitants.

The network used for our simulations was generated by creating a directed and weighted link between any two nodes, *i* and *j*, representing the commuters home location and a work location. The link weight, $w_{ij}^{c}$, represents the total number of commuters who travel every day on that connection for work or study reasons. Every individual older than 3 years is considered a student and included in the network. The original data resolution was at the level of *commune*, however, their number being in the order of 30 000, we coarse-grained the data at level of *arrondissement* (district). Overseas regions and territories of France are excluded from the analysis. Then, the final census commuting network had 329 nodes and 38 077 weighted links for a total 8 019 636 commuters.

The mobile phone commuting network is extracted from a 2007 mobile phone billing information dataset, including 5 695 974 subscribers. This dataset provides temporal and spatial information of user activity, that is the time of every placed call and the coordinates of the tower-cell from which a call has been placed. Following previous work [36], we identify a user’s home place as the most frequently visited tower-cell in terms of placed calls, and his/her workplace as the second most visited tower. In this process, we considered only users who placed more than 100 calls over the course of the 10 months covered by our dataset. To make the census commuting network and the mobile phone commuting network comparable, we coarse-grained the latter from the tower-cell resolution to the district resolution, following the procedure described in [36], thus mapping the mobile phone network to the same geography of the census network.

As expected, the number of users that are estimated to live in each district is affected by a sampling bias owing to the operator coverage that is not uniform from district to district [36]. To refer the mobile phone network to the same population of the census network, that is the whole French population, we adopted a simple normalization approach. We rescaled the population of each district in the mobile phone network by the population sampling ratio $n_{i}^{\mathrm{mp}}/n_{i}^{c}$, where $n_{i}^{\mathrm{mp}}$ is the resident population of district *i* tracked by the mobile phone dataset and $n_{i}^{c}$ is the resident population of district *i* reported by census. Accordingly, each weight *w*^mp^_*ij*_ of the mobile phone network is rescaled by the same factor. With the chosen normalization, the total population assigned to each node of the network (including commuters and non-commuters) is equal in the two systems, whereas the relative fraction of commuters is larger in the mobile phone network [36]. The final mobile phone commuting network has 329 nodes, 60 817 weighted links and a total of 18 750 497 commuters.

### 2.2. Epidemic metapopulation model

We considered a reaction–diffusion (RD) metapopulation model to simulate epidemic spreading on the commuting networks. The metapopulation model is a spatially structured model where the whole population is divided into sub-populations connected by mobility fluxes. In our study, we considered the population of continental France (*N*=63 201 782) structured into sub-populations corresponding to the 329 districts connected by commuting flows.

The reaction process, describing the local disease transmission, takes place in each node of the network where individuals are assumed to be in homogeneous mixing. We consider a rapidly transmitted infection, such as an influenza-like-illness (ILI), whose spatial spread was found to correlate with commuting movements [39,40]. The natural history of the disease is described by a SIR compartmental model with no demography, in which each individual can be either susceptible (S), infectious (I) or recovered/removed (R). Individuals in the recovered compartment develop a lifelong immunity and can not be infected. Transitions from one state to another are ruled by two parameters: the spreading rate *β* and the recovery rate *μ*. The epidemic model is characterized by the basic reproductive number *R*_0_=*β*/*μ*, that defines the average number of infected individuals generated by one infectious individual in a fully susceptible population, thus leading to the threshold condition *R*_0_>1 for an outbreak in a single population [41].

The diffusion process which drives the disease transmission across the system is mediated by the directed and weighted connections of the commuting network. No other type of movement is considered. The RD process is time-discrete and the dynamics is separated into two components, corresponding to two parts of the commuting day: *work time* when commuters are in their working district and *home time* when commuters are in their home districts. The commuters can be infected in their workplace during *work time* and then spread the disease once they travel back to their home district during *home time* or vice versa. Infectious individuals are allowed to commute. For the sake of simplicity, we do not consider different degrees of severity of clinical symptoms and potentially associated behavioural changes. Each day of a simulation is considered as a typical working day, therefore no weekends or holidays are introduced into the model. We define the number of susceptible, infected and recovered who live in district *i* and work in district *j* as *S*_*ij*_, *I*_*ij*_ and *R*_*ij*_, respectively.

At each time step, the number of new infected individuals in each node is extracted from a binomial distribution with a number of trials equal to the number of susceptible individuals in that node, and probability of success equals the force of infection *λ*_*i*_ of the node. The force of infection and the number of infected individuals in each node vary in time and depend on the part of the day we are considering. We define the force of infection during home time as $\lambda_{i}^{h}$ and during work time as $\lambda_{i}^{w}$.

They can be expressed as

2.1 $$\lambda_{i}^{h}=\beta \frac{I_{ii}+\sum_{j\in \nu_{i}}I_{ij}}{N_{ii}+\sum_{j\in \nu_{i}}N_{ij}}$$

and

2.2 $$\lambda_{i}^{w}=\beta \frac{I_{ii}+\sum_{j\in \nu_{i}}I_{ji}}{N_{ii}+\sum_{j\in \nu_{i}}N_{ji}},$$

where *N*_*ij*_=*S*_*ij*_+*I*_*ij*_+*R*_*ij*_ is the total population of commuters living in *i* and working in *j*, while *N*_*ii*_ are the residents of *i* who do not commute. The number of susceptible individuals that are present in node *i* during home time and work time can be computed as

2.3 $$S_{i}^{h}=S_{ii}+\sum_{j\in \nu_{i}}S_{ij}$$

and

2.4 $$S_{i}^{w}=S_{ii}+\sum_{j\in \nu_{i}}S_{ji},$$

where the sums run over the neighbourhood of node *i* : *j*∈*ν*_*i*_.

### 2.3. Numerical simulations and data analysis

We systematically considered each of the 329 network nodes as the initial seed of simulated outbreaks and for each seed we ran 1000 stochastic realizations on both networks, thus resulting in 658 000 simulated epidemics. Throughout our study, we model an ILI transmission characterized by an exponentially distributed infectious period with average *μ*^−1^=3 days. We chose a value of *β*, such that the local basic reproductive number is set to the constant value *R*_0_=1.5 for all simulations. For such value of *R*_0_, the whole system is above the epidemic threshold and the probability of generating a global outbreak is close to 1 for every simulated epidemic [42]. Each simulation is initialized with a number of infected individuals in the seed node equal to 10 and it is run until the epidemic stops spreading across the network (*I*_*i*_=0, ∀*i*). As output of each stochastic simulation we considered: (i) the *arrival time* *t*_*i*_ of the infection in node *i*, defined as the first time step an infected individual is recorded in a fully susceptible subpopulation; (ii) the daily *incidence* in each node, defined as the number of new infected at every time step; (iii) the daily *prevalence* in each node, defined as the number of total infected at every time step; and (iv) the *epidemic infection path* which specifies the disease progression in space by defining a directed link *i*→*j* from the infecting to the infected subpopulation [43].

To compare the temporal diffusion in the two networks, we computed the average arrival time in each sub-population *i* over the 1000 model realizations for a given seed in the mobile phone network $\left\langle t_{i}^{M}\right\rangle$ and in the census network $\left\langle t_{i}^{C}\right\rangle$. The Spearman rank correlation coefficient *r*_*s*_ between $\left\langle t_{i}^{M}\right\rangle$ and $\left\langle t_{i}^{C}\right\rangle$ was computed to measure the strength of monotonic relationship between the mean arrival times in the two networks, for each seed *s*. It is worth recalling that mobility networks extracted from mobile phone data systematically overestimated the commuting fluxes with the considered normalization, therefore accelerating the speed of invasion of the disease [36]. To discount the systematic difference in arrival times owing to such bias, we used a non-parametric measure to focus on the temporal ordering of the infected sub-populations.

To investigate the spatial spread of simulated epidemics, we built the infection path for each model realization and then we computed the minimum spanning tree of all the infection paths originating from the same outbreak seed [43]. The infection path is built by adding a directed and weighted link between node *i* and node *j* when node *i* has seeded the infection in node *j*. The weight represents the fraction of simulations in which the seeding event was observed on that link. The minimum spanning tree extrapolates the most likely transmission route of the infection for a given epidemic scenario, i.e. for a given seed, by minimizing the weighted distance between the origin node and all the other nodes of the network. We then compared the similarity of spatial epidemic patterns by computing the Jaccard index of the minimum spanning trees. For each different outbreak seed *s*, the Jaccard index is defined as

2.5 $$J(s,M,C)=\frac{\left\{M\}\cap \{C\right\}}{\left\{M\}\cup \{C\right\}},$$

where {*M*} represents the set of links in the minimum spanning tree of the mobile phone network epidemics and {*C*} is the set of links in the minimum spanning tree of the census network.

### 2.4. Network features

To identify the characteristics of the outbreak seed that were most likely to generate similar epidemic patterns on the two networks, we used the Pearson correlation coefficient to correlate all the values of *r*_*s*_ and *J*(*s*,*M*,*C*) with a number of centrality measures characterizing the seed *s*. More specifically, we considered the following features measured on both mobility networks and labelled as *M* and *C* to identify the mobile phone network and the census network, respectively. First, the node degree *k*_*i*_, defined as the number of edges in the graph that are incident on node *i* and that can be ingoing, outgoing or the sum of the two.

Second, the node strength or traffic, *T*_*i*_ (ingoing, outgoing and total) defined as

2.6 $$\begin{array}{rl} T_{i,\mathrm{in}} & =\sum_{j\in \nu_{i}}w_{ij}, \end{array}$$

2.7 $$\begin{array}{rl} T_{i,\mathrm{out}} & =\sum_{j\in \nu_{i}}w_{ji} \end{array}$$

2.8 $$\begin{array}{rl} \;\mathrm{and}\;T_{i,\mathrm{tot}} & =T_{i,\mathrm{in}}+T_{i,\mathrm{out}}. \end{array}$$

We also considered various combinations of these quantities to quantify differences between the networks:

— absolute difference in degree: $|k_{x}^{C}-k_{x}^{M}|$;
— relative difference in degree: $|k_{x}^{C}-k_{x}^{M}|/k_{x}^{C}$;
— absolute difference in traffic: $|T_{x}^{C}-T_{x}^{M}|$; and
— relative difference in traffic: $|T_{x}^{C}-T_{x}^{M}|/T_{x}^{C}$;

where *x* can be ingoing, outgoing or total. As an additional measure of similarity, we analysed the local network topology of each seed by means of the *loyalty* [44]. The loyalty *Θ* is a quantity that measures the fraction of preserved neighbours of a node in the two networks. If we define $V_{i}^{C}$ as the set of neighbours of node *i* in the census network and $V_{i}^{M}$ as the same set in the mobile phone network, then $\Theta_{i}^{C,M}$ is given by the Jaccard index between $V_{i}^{C}$ and $V_{i}^{M}$:

2.9 $$\Theta_{i}^{C,M}=\frac{V_{i}^{C}\cap V_{i}^{M}}{V_{i}^{C}\cup V_{i}^{M}}.$$

Loyalty takes values in the interval [0,1], with *Θ*=0 indicating that no neighbours are retained, and *Θ*=1 that exactly the same set of neighbours is preserved. Given that the networks under study are directed, loyalty can be measured on the ingoing, outgoing and complete set of neighbours.

Eventually, we compared our results against node variables that are not directly related to the network structure, such as the node geographical coordinates (longitude and latitude), the node population, the local mobile operator coverage (expressed as a population fraction) and the median income I of the node population (2012 data, available from [45]).

### 2.5. Model evaluation and selection

To identify which network feature or set of features was the best predictor of the similarity of spatial and temporal epidemic patterns, we extensively examined the predictive performance of a linear model which included all the combinations of variables that alone were found to be positively correlated with *r*_*s*_ or *J*(*s*). Specifically, we measured the predictive power of the multiple linear regression in the form

2.10 Y=αX,

where the response vector ***Y*** represents the values *r*_*s*_ or *J*(*s*) for each seed *s*, and the predictors ***X*** are chosen among all the possible combinations of 17 variables: population *P*, degree $k_{x}^{M,C}$, traffic $T_{x}^{M,C}$, loyalty *Θ*_*x*_ and median income I. To compare the performance of the linear models for each response variable, we computed the Akaike information criterion (AIC), where smaller values of AIC indicate a better quality of the model, and we measured the difference $\Delta \text{AIC}={\text{AIC}}_{i}-{\text{AIC}}_{\min}$ [46]. Given the known limitations of a stepwise selection [47], we evaluated all the possible combinations of the selected covariates independently, thus considering all the resulting 131 055 linear models, and compared them with the AIC.

## 3. Results

**Figure 1.**
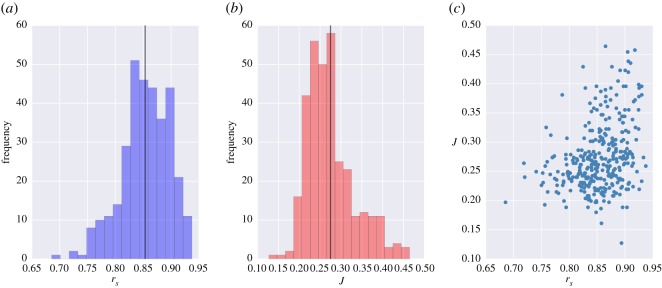
Distributions of similarity measures of epidemic simulations and their correlation. Frequency distributions of the Spearman’s rank correlation coefficient measured between arrival times on the two networks (*a*) and the Jaccard similarity index between the infection trees on the two networks (*b*). Each value of *r*_*s*_ and *J*(*s*) is computed over a statistical ensemble of 1000 simulations for a given outbreak seed *s*. Both histograms correspond to 329 binned values, one for each node of the commuting networks, and solid lines indicate the average of the distributions. Panel (*c*) shows the relationship between *J*(*s*) and *r*_*s*_ for each node of the networks.

The degree of similarity in terms of temporal and spatial unfolding of the simulated epidemics displayed a high variability across the two networks, census and mobile phone, depending on the nodes that were selected as a seed of the outbreak. In general, the temporal hierarchy of the epidemics, measured by the Spearman’s rank correlation *r*_*s*_ between the arrival times of all the nodes, was found to be very similar between the two networks with *r*_*s*_>0.69 for every seed *s* of the network. Figure 1*a* shows that the distribution of *r*_*s*_ ranges between *r*_*s*_=0.69 and *r*_*s*_=0.94, with average ${\bar{r}}_{s}=0.85$. The similarity between the spatial infection patterns on the two networks was more widely distributed with values of the Jaccard index ranging between *J*=0.13 and *J*=0.46, with average $\bar{J}=0.27$ (figure 1*b*). Therefore, while the temporal sequence of infected nodes during an outbreak was generally well preserved in the two networks for most of the epidemic seeds, the paths of infection varied significantly with as few as 13% of transmission links shared between infection trees in some scenarios. Figure 1*c* highlights how the similarity of temporal patterns measured by *r*_*s*_ was only mildly correlated with the spatial similarity measured by *J* (*ρ*=0.33). The two quantities displayed a different behaviour and high values of *r*_*s*_ were sometimes associated to low values of *J*, showing that they provide a different view of the system under study.

The similarity of both spatial and temporal epidemic patterns was mainly driven by a few features of the outbreak seed that were in general positively correlated with each other, specifically the degree of a node and its traffic. As shown in table 1, the Pearson’s correlation coefficient *ρ* between all the degree measures and the similarity of temporal (*r*_*s*_) and spatial patterns (*J*(*i*,*M*,*C*)) displayed a significant positive value, ranging between 0.425 and 0.527 for the invasion sequences and between 0.661 and 0.765 for the invasion trees. Likewise, all the traffic measures of the outbreak nodes were positively correlated with the similarity of epidemic patterns (table 2) with smaller but still significant values of the Pearson’s coefficient, *ρ*=[0.246−0.427] for the temporal diffusion and *ρ*=[0.408−0.588] for the spatial diffusion. Node population, which is generally expected to correlate with degree and traffic, was also found to be a significant predictor for the similarity of epidemic temporal (*ρ*=0.403) and spatial patterns (*ρ*=0.687) as shown in table 3. The median income was weakly correlated with *r*_*s*_ and *J* (*ρ*=0.168 and *ρ*=0.356). Other geographical and demographic variables were not significantly correlated with the similarity of epidemic patterns.

**Table 1.** Similarity of simulated epidemics patterns as a function of the degree of the outbreak seed.

	temporal diffusion		spatial diffusion	
variable	Pearson coefficient *ρ*	*p*-value	Pearson coefficient *ρ*	*p*-value
*k*^C^_in_	0.425	*p*<10^−5^	0.759	*p*<10^−5^
*k*^M^_in_	0.438	*p*<10^−5^	0.661	*p*<10^−5^
*k*^C^_out_	0.527	*p*<10^−5^	0.723	*p*<10^−5^
*k*^M^_out_	0.476	*p*<10^−5^	0.684	*p*<10^−5^
*k*^C^_tot_	0.480	*p*<10^−5^	0.765	*p*<10^−5^
*k*^M^_tot_	0.462	*p*<10^−5^	0.680	*p*<10^−5^
$|k_{\mathrm{in}}^{C}-k_{\mathrm{in}}^{M}|$	−0.042	0.45	−0.253	*p*<10^−5^
$|k_{\mathrm{out}}^{C}-k_{\mathrm{out}}^{M}|$	0.141	0.01	0.262	*p*<10^−5^
$|k_{\mathrm{tot}}^{C}-k_{\mathrm{tot}}^{M}|$	0.041	0.45	−0.066	0.23
$\frac{|k_{\mathrm{in}}^{C}-k_{\mathrm{in}}^{M}|}{k_{\mathrm{in}}^{C}}$	−0.282	*p*<10^−5^	−0.437	*p*<10^−5^
$\frac{|k_{\mathrm{out}}^{C}-k_{\mathrm{out}}^{M}|}{k_{\mathrm{out}}^{C}}$	−0.349	*p*<10^−5^	−0.303	*p*<10^−5^
$\frac{|k_{\mathrm{tot}}^{C}-k_{\mathrm{tot}}^{M}|}{k_{\mathrm{tot}}^{C}}$	−0.339	*p*<10^−5^	−0.469	*p*<10^−5^

**Table 2.** Similarity of simulated epidemics patterns as a function of the traffic of the outbreak seed.

	temporal diffusion		spatial diffusion	
variable	Pearson coefficient *ρ*	*p*-value	Pearson coefficient *ρ*	*p*-value
*T*^C^_in_	0.246	*p*<10^−5^	0.408	*p*<10^−5^
*T*^M^_in_	0.331	*p*<10^−5^	0.518	*p*<10^−5^
*T*^C^_out_	0.409	*p*<10^−5^	0.511	*p*<10^−5^
*T*^M^_out_	0.427	*p*<10^−5^	0.588	*p*<10^−5^
*T*^C^_tot_	0.317	*p*<10^−5^	0.466	*p*<10^−5^
*T*^M^_tot_	0.380	*p*<10^−5^	0.560	*p*<10^−5^
$|T_{\mathrm{in}}^{C}-T_{\mathrm{in}}^{M}|$	0.443	*p*<10^−5^	0.620	*p*<10^−5^
$|T_{\mathrm{out}}^{C}-T_{\mathrm{out}}^{M}|$	0.391	*p*<10^−5^	0.605	*p*<10^−5^
$|T_{\mathrm{tot}}^{C}-T_{\mathrm{tot}}^{M}|$	0.451	*p*<10^−5^	0.667	*p*<10^−5^
$\frac{|T_{\mathrm{in}}^{C}-T_{\mathrm{in}}^{M}|}{T_{\mathrm{in}}^{C}}$	−0.421	*p*<10^−5^	−0.258	*p*<10^−5^
$\frac{|T_{\mathrm{out}}^{C}-T_{\mathrm{out}}^{M}|}{T_{\mathrm{out}}^{C}}$	−0.468	*p*<10^−5^	−0.140	0.01
$\frac{|T_{\mathrm{tot}}^{C}-T_{\mathrm{tot}}^{M}|}{T_{\mathrm{tot}}^{C}}$	−0.562	*p*<10^−5^	−0.295	*p*<10^−5^

**Table 3.** Similarity of simulated epidemics patterns as a function of non-network variables.

	temporal diffusion		spatial diffusion	
variable	Pearson coefficient *ρ*	*p*-value	Pearson coefficient *ρ*	*p*-value
population	0.403	*p*<10^−5^	0.687	*p*<10^−5^
median income	0.168	0.0002	0.356	*p*<10^−5^
coverage	0.018	0.75	0.007	0.91
latitude	0.283	*p*<10^−5^	0.054	0.33
longitude	−0.176	0.001	−0.055	0.32

**Figure 2.**
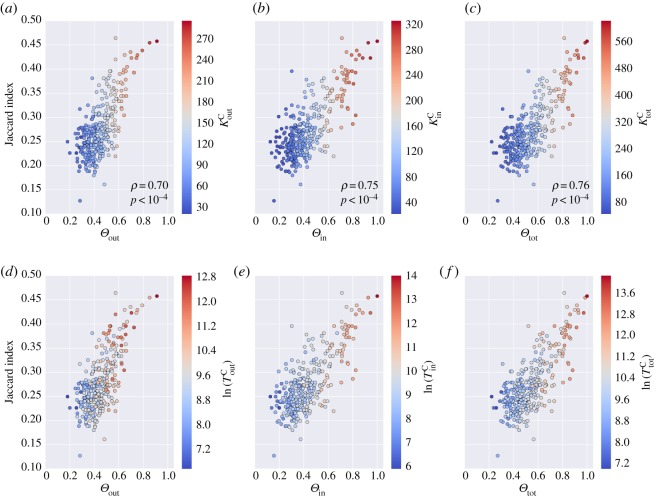
Comparing the similarity of invasion trees and loyalty. Each panel shows the Jaccard similarity index measured between the epidemic infection tree of the census network and the infection tree of the mobile phone network against the loyalty of the seed node: (*a*,*d*) *Θ*_in_, (*b*,*e*) *Θ*_out_, (*c*,*f*) *Θ*_tot_. Points are scatter plot for each node of the network that seeded the epidemic. Colour gradient, from blue to red, represents increasing values of (*a*) *K*^C^_out_, (*b*) *K*^C^_in_, (*c*) *K*^C^_tot_, (*d*) *T*^C^_out_, (*e*) *T*^C^_in_, (*f*) *T*^C^_tot_. Traffic values are shown on a log scale.

**Figure 3.**
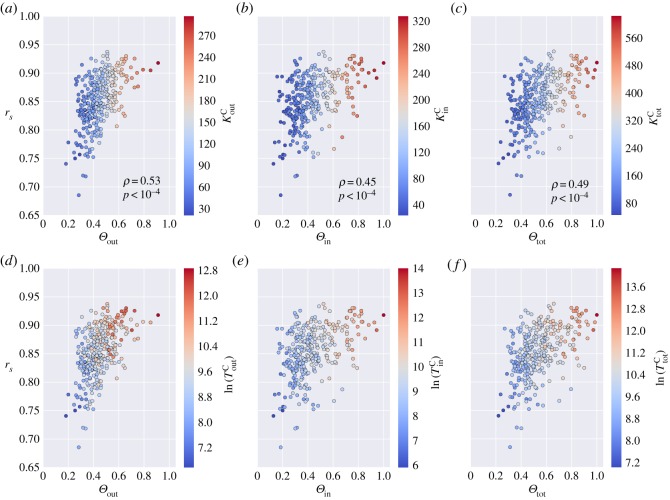
Comparing the correlation of arrival times and loyalty. Each panel shows the Spearman’s correlation coefficient measured between the arrival times on the census network and the mobile phone network against the loyalty of the seed node: (*a*,*d*) *Θ*_in_, (*b*,*e*) *Θ*_out_, (*c*,*f*) *Θ*_tot_. Points are scatter plot for each node of the network that seeded the epidemic. Colour gradient, from blue to red, represents increasing values of (*a*) $K_{\mathrm{out}}^{C}$, (*b*) *K*^C^_in_, (*c*) *K*^C^_tot_, (*d*) *T*^C^_out_, (*e*) *T*^C^_in_, (*f*) *T*^C^_tot_. Traffic values are shown on a log scale.

Overall the above centrality measures were significantly correlated with the node loyalty, indicating that incoming and outgoing mobility flows of the census network were better captured by CDR data for highly connected, busy and highly populated locations. Figure 2 summarizes this result by showing scatter plots of the epidemic tree Jaccard index *J* and the outgoing (figure 2*a*,*d*), ingoing (figure 2*b*,*e*) and total (figure 2*c*,*f*) loyalty *Θ* of the outbreak seed. In each panel, dots are colour coded according to the degree (figure 2*a–c*) or traffic (figure 2*d–f*) of the seed node. It clearly appears that the higher the loyalty of the outbreak seed, the higher the similarity of the final infection trees. The Pearson’s correlation coefficient between the two quantities varies between *ρ*=0.7 for *Θ*_out_ and *ρ*=0.76 for *Θ*_tot_. Moreover, the colour gradient indicates that *Θ*_out_, *Θ*_in_ and *Θ*_tot_ are positively correlated with all the measures of degree and traffic considered. Along the same lines, the loyalty was a significant predictor of the invasion chronology (figure 3) with the Pearson’s correlation coefficient between *r*_*s*_ and *Θ* ranging from *ρ*=0.45 for *Θ*_out_ to *ρ*=0.53 for *Θ*_tot_.

When looking at the absolute differences in the seed degree between the two mobility networks, the correlation with the epidemic patterns was in general not significant (table 1). Instead, the absolute difference in traffic was found to be a good predictor of the epidemic patterns’ similarity (table 2) with a positive correlation *ρ*=[0.391−0.667]. Shifting our attention from absolute to relative differences in degree and traffic, these were found to be negatively and significantly correlated with the accordance of epidemic patterns, both for the invasion chronology (*ρ*=[−0.562–0.282]) and the invasion trees (*ρ*=[−0.469–0.140]). This can be explained by the fact that epidemics seeded in peripheral nodes were the most affected by discrepancies in the two networks and most likely to display a different epidemic pattern from one model to the other.

Moving from the analysis of nodes’ features taken one by one to a multiple linear regression based on a set of predictors, the best models were all found to be a linear combination of several variables (table 4). Specifically, the best linear model when ***Y***≡*r*_*s*_ was found to be a combination of 10 variables and the best linear model for ***Y***≡*J*(*s*), was a multiple linear regression of seven variables. Compared to the best model with ${\text{AIC}}_{\min}$, we found 30 and 45 models to be within the range *Δ*AIC<2, which represents a traditional threshold for model selection [46]. Imposing a threshold *Δ*AIC<4, we would have selected 255 models for ***Y***≡*r*_*s*_ and 492 models for ***Y***≡*J*(*s*).

**Table 4.** Multiple linear regressions. (The two models with minimum AIC are shown only.)

model	variables	AIC
*r*_*s*_=*α****X***	*P*, *K*^C^_in_, *K*^C^_tot_, *T*^C^_in_, *T*^C^_out_, *T*^M^_in_, *T*^C^_tot_, *Θ*_in_, *Θ*_out_, I	−1338.43
*J*=*α****X***	*P*, *K*^C^_tot_, *T*^C^_in_, *T*^C^_out_, *T*^C^_tot_, *T*^M^_tot_, *Θ*_out_	−1248.88

## 4. Discussion

In this study, we systematically evaluated the goodness of a large-scale mobile phone dataset to represent commuting movements in France, as reported by the official census, when integrated into a spatially structured epidemiological model. Overall, the mobile phone network was found to represent more faithfully the commuting links originating from or incoming to the most connected locations, which also turn out to be the busiest and most populated, as demonstrated by the correlation between node loyalty and its degree and traffic. As the infection tree of the epidemics is structurally defined by the topology of the underlying mobility network [48], the similarity of spatial epidemic patterns was found to be best explained by the loyalty of the seed node. This result suggests that obtaining an accurate description of the local connectivity around the origin of the outbreak could be sufficient to capture spreading patterns on a larger spatial scale.

The chronology of the epidemic invasion was in general well preserved on both mobility networks and showed a milder dependence on the characteristics of the outbreak seed. This suggests that the arrival order of an epidemic can be well predicted also considering a proxy for mobility such as mobile phone data, in agreement with the known fact that the arrival times distribution in a metapopulation model can be estimated by measuring a certain weighted distance computed on a directed weighted graph [49]. Still, the agreement between temporal sequences of infection on the two networks was significantly correlated to the degree, the traffic and, ultimately, to the loyalty of the seeding node, confirming a better match for epidemics seeded in central locations.

Results confirmed the initial findings of our previous work [36], where, based on a limited exploration of different initial conditions, connectivity and traffic of the seed node were found to contribute to the similarity across networks. On the other hand, the multiple regression analysis showed that almost all features of the outbreak seed contributed to shaping the epidemic patterns and their similarity, to some extent. For both temporal and spatial similarity, the best predictive models were based on several variables and hundreds of models were found to be statistically equivalent based on their AIC values. It was not possible, therefore, to identify a parsimonious set of characteristics of the seed node that alone would best predict the epidemic outcomes on both networks. Indeed, designing a set of rules to extrapolate the results of simulated epidemics from a proxy mobility network to another one, and possibly generalizing such rules across different countries, would be of highly practical importance. However, our results suggest that a perfect match between simulated epidemics on two mobility networks cannot be obtained by a simple rescaling or normalization based on a single or few network metrics.

We focused uniquely on one-type human movement, that is daily commuting, for several reasons: it has been shown to be relevant for the spread of influenza at the national level [39], it is accurately recorded by census surveys for the whole population with very limited sampling bias, epidemic models based on recurrent mobility have been tested on real disease data [50,51], and finally, home-work movements can be extracted from mobile phone data with very good precision for almost every user [52]. In our case, both datasets referred to the same year and the definition of commuting in the census data included both home-work and home-school movements, thus virtually representing the best available description to be matched with mobile phone data. Extending our analysis to include all types of movement into an epidemic model can be challenging owing to the lack of data from census or travel surveys on a geographical scale large enough to be compared with mobile phone records. Other studies have compared human movement data from CDRs and travel or migration surveys in a low-income setting [35,37], nonetheless both were either limited to a few thousand individuals in a relatively small region for travel surveys or by the low resolution in the migration data.

In our study, we considered a best case scenario where the two mobility datasets could be aligned to a high degree. An even better match could be obtained by refining the normalization procedure and by identifying more accurately home and work locations by including more metadata, as tested in [36]. However, we do not expect these refinements to impact significantly on our results, as they will introduce second order changes in the topology of the mobile phone network. On the other hand, in most real situations CDRs will be affected by geographical biases in network coverages and usage or incurred by certain demographic groups being more frequent phone users [53], not to mention how difficult the access to such data can be owing to their sensitive nature. Trying to correct for such socio-economic biases, by taking them into account in the normalization procedure, could provide a potential explanation for the discrepancies observed between epidemic outcomes on the two networks. On the other hand, it would also increase the number of parameters or assumptions in the model. For this reason, we decided to focus on a basic normalization, as it is also more easily generalizable to other settings where socio-economic variables are not easily accessible. Also, alternative sources of mobility have been proposed to complement mobile phone records such as GPS [54] or social media traces [55,56], the latter being more easily accessible at the cost of introducing other uncertainties on the demographics of the travellers and the type of movement.

Here, we focused on modelling an ILI. Additional heterogeneities and factors might be relevant to realistically capture the spatial spread of ILIs [57], here however we adopted minimal modelling assumptions that allowed us to clearly isolate the effects of different mobility networks on the disease spread. Results might be different when considering other diseases for which movements other than commuting might be relevant, and for which transmission is driven by environmental conditions, such as cholera or poliovirus. In general, we expect our findings to hold true within the modelling of rapidly disseminated directly transmitted infections.

A significant amount of research in the past decade has clearly highlighted the fact that one single source of mobility data cannot provide a full and comprehensive description of human movements across all spatio-temporal scales that are relevant for infectious disease transmission. Moreover, there is no *a priori* correct level of aggregation for analysis of human mobility and infectious disease dynamics [58]. It becomes clear that, to obtain a detailed picture of human mobility in an area for epidemic modelling, it is necessary to consider combinations of data in a multilayer fashion [21,59]. To what extent modelling results are affected by the integration of one particular mobility proxy compared to others, and how this relates to the epidemiological properties of the disease under study—whether it could be a rapidly transmitted infection or a vector borne disease—requires further research.

Our approach compared mobile phone data to infer recurrent mobility flows as reported by census surveys in France to be then integrated into a metapopulation model for ILIs. Results suggest that obtaining an accurate description of human movements in the area at the origin of the outbreak can be essential to capture its future spreading patterns, and that mobile phones are more reliable in central regions than peripheral ones. However, it would be important to investigate how this requirement can be reduced by changing the spatial resolution of interest and how this depends on the use of mobile phone data or other proxies to approximate human mobility. Continued work along these directions is important to understand how to measure epidemiologically relevant patterns of movement to be further integrated into computational models which can ultimately help in forecasting and controlling disease spread.

## Data accessibility

The commuting networks are available as electronic supplementary material files of [36].
